# CRX haploinsufficiency compromises photoreceptor precursor translocation and differentiation in human retinal organoids

**DOI:** 10.1186/s13287-023-03590-3

**Published:** 2023-12-05

**Authors:** Deng Pan, Xiao Zhang, Kangxin Jin, Zi-Bing Jin

**Affiliations:** Beijing Institute of Ophthalmology, Beijing Tongren Hospital, Capital Medical University, Beijing, 100730 China

**Keywords:** CRX, Haploinsufficiency, Photoreceptor, Human, Retinal organoids, Development, Inherited, Mutation, Disease

## Abstract

**Background:**

The *CRX*-associated autosomal dominant retinopathies suggest a possible pathogenic mechanism of gene haploinsufficiency. However, based on reported human patient cases and studies with mouse models, it is hard to confirm the specific weight of haploinsufficiency in pathogenesis due to the interspecies gaps between gene expression and function.

**Methods:**

We created monoallelic *CRX* by replacing one allele with tdTomato in human embryonic stem cells (hESCs) and subsequently dissect pathogenesis in hESCs-derived retinal organoids. We used transcriptome and immunofluorescence analyses to dissect phenotypic differences between *CRX*-monoallelic knockout and control wildtype organoids. For location analysis of *CRX*^+^ cells, a *CRX*-expression-tracing system was constructed in control hESCs. We implemented long-term live-cell imaging to describe the translocation of CRX^+^ cells between two groups in early organoid differentiation. The expression pattern of these dynamic differences was validated using RNA-seq and immunofluorescence assays.

**Results:**

We identified delayed differentiation of outer nuclear layer (ONL) stratification along with thinner ONL, serious loss of photoreceptor outer segments, as well as downregulated expression of gene for phototransduction and inner/outer segment formation. By live-cell imaging and immunostaining, we observed the overtension of actomyosin network and the arrested translocation of monoallelic *CRX*^+^ cells in the early stage of retinal differentiation.

**Conclusions:**

We confirmed that gene haploinsufficiency is the mechanism for the dominant pathogenicity of *CRX* and discovered that CRX regulated postmitotic photoreceptor precursor translocation in addition to its specification of photoreceptor cell fates during human retinal development. These findings revealed a new underlying mechanism of CRX dominant pathogenesis and provided a new clue for the treatment of *CRX*-associated human retinopathies.

**Supplementary Information:**

The online version contains supplementary material available at 10.1186/s13287-023-03590-3.

## Introduction

Autosomal dominant inherited diseases are associated with complicated pathogenic mechanisms, including haploinsufficiency, gain-of-function, and dominant negative, thus bringing huge challenge. About 80 genes have been reported in inherited retinal dystrophy (RetNet: https://web.sph.uth.edu/RetNet/) [1], and many of them possessed obscure pathogenic mechanism. An expanded thought with more dimensions of measurements including molecular, cellular, and behavioral tests is urgently needed to guide the pathogenicity dissection, on basis of which may provide more understanding of etiology and help us to formulate more refined treatment strategies to combat disease progression.

Cone-rod homeobox (CRX) is a transcription factor that is vital in regulation of retinal development and photoreceptor maintenance [2–4]. By coordinating with other photoreceptor genes, rod and cone cell fate specification and maturation are determined for receiving and transducing optical signals [5–13]. There are a large number of CRX binding domains in human and mouse photoreceptor cells, including genes with mutations leading to retinal diseases, as well as related factors involved in the transcriptional network of rods and cones [14–17]. Interestingly, CRX is a well-known marker of retinal progenitor cell mitosis exit and is expressed early in retinal development; however, its role beyond the specification of photoreceptor cell fates remains to be elucidated.

CRX was the first photoreceptor transcription factor identified to cause retinal degeneration. To date, approximately 100 *CRX* mutations have been identified to cause dominant retinal degeneration with varied severity in clinical phenotype, including Macular Dystrophy, Retinitis Pigmentosa, Cone-Rod Dystrophy and Leber Congenital Amaurosis [18, 19]. Mutations are widely scattered in different domains; no unified conclusion can correlate the severity of *CRX*-associated retinopathies with the mutation spectrum [20]. Current studies suggest that dominant negative and/or haploinsufficiency, as well as recessive or multigenic mechanisms, contribute to *CRX* mutation-allied disease. The complex situation makes the treatment strategy designed only in the direction of heterogeneity, which is difficult to be applied in practice. Clarifying the specific weight of haploinsufficiency in pathogenesis will help simplify the situation and determine whether gene augmentation should be a major consideration. Unfortunately, few cases of complete or nearly complete deletion of monoallelic of *CRX* have been reported, and parents and offspring exhibit different phenotype ranging from unaffected to poor vision, making the ambiguous judgment of haploinsufficiency [21–24]. In contrast, mice with monoallelic deletion exhibited a much milder phenotype with a recoverable loss of outer segments (OSs) and ERG amplitude. In fact, *Crx*-deficient mice carrying human mutation does not always linearly reproduce the human clinical phenotype [25, 26]. A model for studying human-specific pathogenesis could therefore be used to address the difficulties.

Stem cell-derived organoids have made great progress in modeling human diseases and in translational researches for therapies [27–32]. Organoid differentiation mimics human embryonic development and has the advantage of being more closely to study human-specific gene transcription than other animal models. The recapitulative process can perfectly fuse gene editing for genetic modification and fluorescent protein labeling and also are compatible with long-term live-cell imaging for dynamic process monitoring [33–35]. In this study, we use human embryonic stem cells (hESCs) to generate retinal organoids (ROs) by mimicking the retinal development after *CRX* monoallelic deletion. We revealed that the monoallelic transcription of *CRX* caused a loss of the photoreceptor OSs and a downregulation of phototransduction related genes, thus demonstrating serious dominant pathogenicity. We found a delayed retinal stratification during differentiation in the ROs. In the early differentiation, an overtension of actomyosin network and compromised postmitotic photoreceptor translocation exhibited in retinal organoids, suggesting that *CRX* is involved in more biological processes during early retinal development.

## Methods

### Generation of tdTomato knock-in hESC lines

**Fig. 1:**
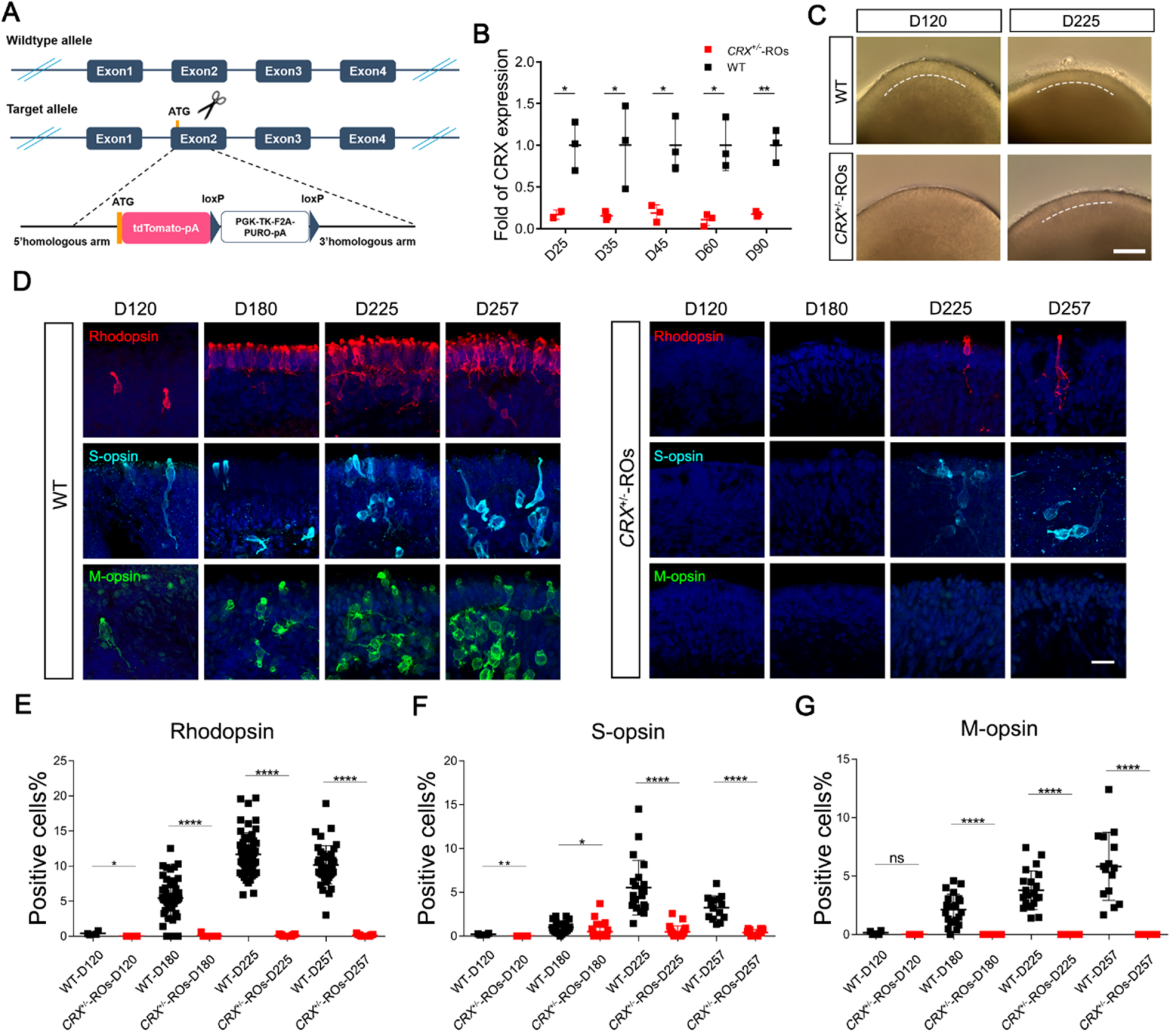
Generation and characterization of *CRX* monoallelic transcription. **A** Schematic diagram of *CRX* monoallelic deletion in H9 ESCs. The encoding ORF of one allele of *CRX* was abolished and replaced with tdTomato gene. **B** The quantitative PCR analysis of relative *CRX* expression in different stage of *CRX*^±^-ROs compared with wildtype ROs. Significance tested with *t*-test: *, *p* < 0.05; **, *p* < 0.01. **C** Bright field images of wildtype and *CRX*^±^-ROs at D120 and D225. Dashed-lines indicated the outer nuclear layer (ONL) stratification on the outer edge of the organoid. Scale bar, 400 μm.** D** Immunostaining of Rhodopsin, S-opsin and M-opsin in D120, D180, D225 and D257 wildtype and *CRX*^±^-ROs. Tissues were counterstained with DAPI (in blue). Scale bar, 20 μm. **E–G** Quantification of Rhodopsin^+^, S-opsin^+^ or M-opsin^+^ cells at the indicated stages in wildtype and *CRX*^±^-ROs. Significance tested with *t*-test: *, *p* < 0.05; **, *p* < 0.01; ****, *p* < 0.0001; ns, not significant

We used CRISPR to generate two gene-edited H9 cell lines in this study. The CRX monoallelic deletion cell line was generated in previous work [36]. In brief, we generated the *CRX*^±^ cell line by inserting tdTomato ORF right after the translation initiation site of *CRX* and therefore replacing one of the *CRX* alleles (Fig. 1A). For the *CRX*^+/+^ reporter cell line, P2A and tdTomato sequences were inserted before stop codon of *CRX* without disrupting its expression (Fig. 4D). To generate the biallelic CRX with tdTomato reporter (Fig. 4D), a vector plasmid was firstly constructed in which sgRNA sequences were designed to target between *CRX* exon 4 and the 3'UTR. The second vector serves as the homologous repairing template containing 5’ and 3’ homologous arms, and a short sequence of 2A peptide, a tdTomato coding sequence and puromycin-resistance selection cassette in between the arms. The resulting vectors were delivered into H9 (WAe009-A, WiCell) by electroporation. The expected clones will be harvested by puromycin-resistance screening and identified by PCR and subjected to Sanger sequencing. The puromycin cassette was then removed by flippase (FLP) recombinase.

### hESCs culturing and differentiation of retinal organoid

All H9 cell lines were cultured in fresh mTeSR-E8 medium (Cat. #05940; Stemcell Technologies, Canada) supplemented with 10 μM Y-27632 to reduce cell death. Retinal organoids were generated through a method described previously [49] with a few modifications. Briefly, hESCs were dissociated using TrypLE Select (Cat. #12,563–011; Life Technology) containing 0.05 mg/ml DNase I (Cat. #11,284,932,001; Roche). Then, cells were reaggregated at a density of 9000 cells/100 μl per well in a low attachment 96-well plate with V-bottom (NOF corporation, Tokyo, Japan) in retinal differentiation medium I that contains G-MEM medium (Cat. #11,710–035; Gibco, United States) supplied with 20% (v/v) KSR, 20 μM Y-27632, 3 μM IWR1e (Cat. #681,669; Merck Millipore, United States), 0.1 mM nonessential amino acids, 1 mM pyruvate, 100 mg/ml streptomycin, 0.1 mM 2-mercaptoethanol, and 100 U/ml penicillin. Half of the medium was replaced with fresh medium at day 6 (D6). From D2 to D18, Matrigel (Cat. #356,231; BD Corning, United States) was added in a final proportion of 1% v/v. At D12, all aggregates were transferred into retinal differentiation medium II that contains G-MEM medium supplied with 10% (v/v) FBS, 100 nM SAG (Cat. #ALX- 270–426-M001; Enzo Life Sciences, United States), 0.1 mM nonessential amino acids, 1 mM pyruvate, 100 mg/ml streptomycin, 0.1 mM 2-mercaptoethanol, and 100 U/ml penicillin. At D18, aggregates were cut into small pieces and maintained with neural retina culture medium that contains DMEM/F12-GlutaMAX medium (Cat. #10,565–018; Gibco, United States) supplied with 10% (v/v) FBS, N2 supplement (Cat. #17,502–048; Gibco, United States), 0.5 mM retinoic acid (Cat. #R2625; Sigma, United States), 12.5 mg/ml taurine (Cat. #T0625; Sigma, Japan), 100 mg/ml streptomycin and 100 U/ml penicillin. Aggregates in different stages were all cultured in humidified incubator at 37 °C with 5% CO_2_. For blebbistatin inhibitor experiment, *CRX*^±^-ROs were separated in petri dishes for the experiment groups and Control. At D40, blebbistatin storage solution (10 mM in DMSO) was added into the petri dishes according to the final concentration. The whole operation was under dark condition. After a week, the medium containing the small molecule was replaced to fresh medium.

### Immunohistochemistry

Retinal organoids were fixed in 4% paraformaldehyde for 30 min at room temperature. Cryosections (14 μm) of organoids were permeabilized with 0.5% Triton X-100 (Cat. #A600198-0500; Sangon Biotech, China) for 20 min and blocked for 1 h at room temperature with 1% BSA and 0.5% Triton X-100 in PBS. Then, sections were incubated in blocking buffer for 12 h at 4 °C with a primary antibody. The primary antibodies used in the study include CRX (Cat. #H00001406-M02; Abnova,1:400), VSX2 (Cat. #sc365519; Santa Cruz, 1:200), OTX2 (Cat. #sc514195; Abcam, 1:200), PAX6 (Cat. #PRB-278P; Covance, 1:300), RAX (Cat. #sc271889; Santa Cruz, 1:200), SOX2 (Cat. #sc-17319; Santa Cruz, 1:200), S-opsin (Cat. #AB5407; Millipore, 1:500), L/M-opsin (Cat. #AB5405; Millipore, 1:500), RHO (Cat. #O4886; Sigma, 1:1000), Recoverin (Cat. #AB5585; Millipore, 1:500), KI67 (Cat. #BD6280947; BD Biosciences, 1:200), SOX9 (Cat. #ab185966; Abcam, 1:250), GFAP (Cat. #HPA056030; Sigma, 1:200), CRALBP (Cat. # ab15051; Abcam, 1:200), pMLC2 (Cat. # 3675S; CST, 1:50). After washing with PBS, the sections were incubated for 1 h with fluorescence-conjugated secondary antibodies such as Alexa Fluor 488 donkey anti-rabbit IgG (Cat. #A-21206; Invitrogen), Alexa Fluor Plus 488 goat anti-mouse IgG (Cat. #A32723; Invitrogen), and Alexa Fluor 594 donkey anti-mouse IgG (Cat. #A21203; Invitrogen). Sections were stained for 10 min with DAPI (Cat. #GD3408; Genview) before imaging.

### Quantitative reverse-transcription PCR

Total RNA was isolated from retinal organoids of several timepoints using TRIzol Reagent (Cat. #15,596,018; Invitrogen) with the manufacturer’s instructions. Then, the cDNA samples, reverse-transcribed from total RNA using M-MLV Reverse Transcriptase (Promega; Cat. #M1705), were processed for quantitative PCR using a Mastermix (FastStart Universal SYBR Green Master [ROX]; Roche) in a real-time PCR system (LightCycler 96 System; Roche, Mannheim, Germany) with the primers specific for CRX (forward primer 5’-TCCAGGGTTCAGGTTTGGTTCA -3’ and reverse primer 5’-GGCAGAGGGGGACTGTAGGA-3’). Expression levels of the gene CRX at different timepoints were normalized to that of the housekeeping gene beta-actin (forward primer 5’-ACTCTTCCAGCCTTCCTTC-3’ and reverse primer 5’- ATCTCCTTCTGCATCCTGTC -3’).

### RNA sequencing and analysis

Total RNA was isolated from retinal organoids of several timepoints using TRIzol Reagent (Cat. #15,596,018; Invitrogen) following the manufacturer’s instructions. A minimum of 2 μg of total RNA obtained was used for library construction with NEBNext Ultra RNA Library Prep Kit (#E7530L, NEB, USA) as recommended. Library sequencing was performed on the Illumina NovaSeq 6000 platform to generate pairwise 150-bp read lengths. All reads were mapped to human genome version hg38 by Hisat2 version 7.5.0 at default settings. FeatureCounts (FC) were used for calculating read counts. DESeq2 was used for DEG analysis. The web-based tool Metascape (https://metascape.org/gp/index.html#/main/step1) and DAVID Bioinformatics (https://david.ncifcrf.gov/) were used for protein–protein interaction analysis and function enrichment analysis of DEGs of RNA-seq.

### Organoid cryosections

Images of organoid cryosections were acquired by a Leica SP8 confocal microscope. Single planes were stacked to generate a maximum projection image with proper brightness and contrast processed by self-contained LAS X.

### Two-photon microscopy

Retinal organoids were fixed in 4% paraformaldehyde for 1 h at room temperature. An amount of 2 μl 100% Matrigel was used firstly to immobilize them onto the bottom of culture dishes. The D34 and D45 retinal organoids were immersed in PBS solution and imaged using a Zeiss LSM 880 two-photon microscope equipped with an objective of W "Plan-Apochromat" 20 × /1.0 DIC M27 (N.A. = 1.0). The tdTomato was excited by a 730 nm wavelength laser with intensity of 4.5%. The 3D image reconstruction was carried out with ZEN software.

### Live-cell imaging

Retinal organoids were collected by a pipette and transferred onto glass-bottom dishes and were firstly immobilized onto the bottom of dishes with 2 μl 100% Matrigel. A coverslip with a fixed weight objective on it was placed on organoids for further immobilization according to a published method [34] with some modifications. The culture dish was returned to the cell culture incubator overnight. Live-cell imaging was captured using a Leica TCS SP8 confocal laser scanning system equipped with temperature and CO_2_ control (Stage Top Incubator, TOKAIHI). The tdTomato signal of retinal organoids was excited by a 568 nm laser with an intensity of 0.2% using a 10 × objective (N.A. = 0.4). A resolution of 1024 × 1024-pixel images was acquired for 50 μm depth by a step of 1.5 μm between each frame. Data were exported by LAS X and imported into ImageJ software for subsequent processing.

### Cell counting

A minimum of three individual retinal organoids from different batches was analyzed for each condition and images used for counting were randomly selected. Quantification of positive cells of immunofluorescent images was performed using the ImageJ software.

### Trajectory tracking

The quality of live-cell images was firstly examined. In general, the first few hours of captured frames with unstable state of organoids would be removed, which may be caused by a change of live-cell culturing environment. For all 50 μm-depth images, continuous 30 μm depth with appropriate cell density and the highest cell definition were selected to make the maximum projection. Further trajectory analysis was done on base of these 2D images using the “TrackMate” plugin in ImageJ. Firstly, the images were first swapped Z and T. Then, calibration settings were done to set scale, image depth and time interval. After that, moving cells were detected with the particle parameters “Estimated blob diameter” set to 20 pixels, “Threshold” set to 1 pixel and other parameters set by default. After performing analysis, the results of “Track statistics” including each fluorescent cell in time duration, path length, distance and straightness information were exported for further analysis. The analysis of total length and distance of the cell migration were performed after the trajectory data were grouped according to the duration of the cell appearance. “TrackScheme” was used to save individual paths and further analysis. For analysis of cell migration direction, track information with a duration of more than 10 frames was selected after importing of individual paths. For the analysis of the straightness of movement, the trajectory data of the fluorescent cells with a duration of more than 10 h were included in comparison.

### Cell distribution

The distribution pattern of tdTomato^+^ cells was measured by the distance from the somal centers to the outer edge of retinal organoid. The process of determining the distance (*d*) is converted into a geometric problem of calculating the distance from the internal point to the fitting circle since retinal organoids present a regular curved outer edge generally. Briefly, after the fluorescent images were imported into the ImageJ software and performed scale conversion, the coordinates of three random points on the outer edge of the organoid were randomly selected to generate a fitting circle with determined radius and the center coordinate of the circle. Then, the “Analysis Particles” tool was used to calculate lineal length between the coordinates of the tdTomato^+^ cells and center of circle. Distance could be obtained by subtracting the line lineal length from the radius. After obtaining all distance values, the maximum value in each sample was used to normalize the other values. The distribution of relative distance values ranged from 0 to 1, with *d* = 1 indicating cells at basal side, and *d* = 0 representing cells at apical side. R language was used for further probabilistic distribution analysis.

### Measurement of ONL thickness

DAPI staining was performed to stain the nucleus before image capturing. The outer nuclear layer (ONL) was delineated in images. At least 10 cryosection images, different organoids were included in each statistical analysis, with each Sects. "Results", "Discussion" positions randomly selected for thickness calculation by ImageJ.

### Flow cytometry

After retinal organoids were collected at several timepoints, the organoids were dissociated into single cells using Trypsin–EDTA (0.25%, Life Technologies) supplemented with 20 μm Y-27632. Immediately after the dissociation reaction being stopped by DPBS containing 10% FBS and 20 μm Y-27632, the cell suspension was diluted to a density of 1 × 10^6^ cells/ml and were examined using a BD FACS Aria II and BD FACS Cytometer (BD Pharmingen) with the WT as tdTomato negative controls. Data analysis was performed using FlowJo software.

### Statistical analysis

Statistical analysis was performed with GraphPad Prism (version 6.01). All values were shown as the mean ± standard deviation (SD). Statistical significance was tested using an unpaired two-tailed *t*-test, except for analyses of cells lengths and distances in live-cell imaging, which were statistically analyzed using the paired *t*-test. Statistically significant differences were defined as *P* < 0.05.

## Results

### *CRX* haploinsufficiency delayed retinal stratification and disrupted photoreceptor pattern

To confirm whether *CRX* haploinsufficiency causes dominant pathogenicity, we first constructed an ESCs (H9) cell line with a *CRX* monoallelic deletion. A N-terminal knock-in strategy was used as previously described [36], the *tdTomato* fragment with polyA-tail was inserted behind *CRX* promoter, encoding to create a modified ESC line with monoallelic transcriptional of *CRX* (Fig. 1A). In the early and middle stages of differentiation, the bright field images of *CRX* monoallelic retinal organoids (*CRX*^±^-ROs) looked like the same as those of wildtype H9-ROs (Additional file 1: Fig. S1A). Immunostaining of early ocular development marker (RAX), retinal progenitor cell markers (SOX2, PAX6, VSX2, OTX2) showed no differences between two groups, indicating that *CRX* monoallelic transcription does not affect the directional differentiation from pluripotent stem cells to retina (Additional file 1: Fig. S1B). Quantitative PCR of *CRX* showed there was a 2-to threefold reduction in *CRX*^±^-ROs compared to control (Fig. 1B), in agreement with the monoallelic deletion of *CRX*.

Differences in organoid morphology do not appear until D120, when control group showed a highlighted reflective ring on the outer edge of the organoid in bright field images, which in our and others' experience indicated the outer nuclear layer (ONL) stratification. The *CRX*^±^-ROs did not show the typical ONL structure highlighted at D120 but at D225, suggesting a delay in ONL stratification. In D225 ROs of control group, the appearance of extended brushes containing the photoreceptor inner segments (ISs) and OSs indicated that the photoreceptor was gradually maturing. However, the D225 *CRX*^±^-ROs contains significantly many fewer outer/inner segments (Fig. 1C), suggesting that the ability to generate inner/outer segments was impaired.

The generation of photoreceptor opsins marks the maturation of photoreceptor cells. We examined opsin expressions in rod and cone cells in two groups (Fig. 1D–G). Rhodopsin, S-opsin and M-opsin, cell markers for rod, blue cone and green cone, respectively, can all be detected at D120 ROs in control group. In contrast, Rhodopsin and S-opsin could not be detected until D180 and D225 in *CRX*^±^-ROs, respectively, and did not increase their positive cell production even if the differentiation time is further prolonged. M-opsin^+^ cells cannot be detected in *CRX*^±^-ROs at all stages. Therefore, *CRX* haploinsufficiency is sufficient to cause destructive photoreceptor disorders in *CRX*^±^-ROs, which is similar to the severe LCA disease phenotype in human.

### Altered gene expressions in ***CRX***^±^ organoids at late differentiation

**Fig. 2:**
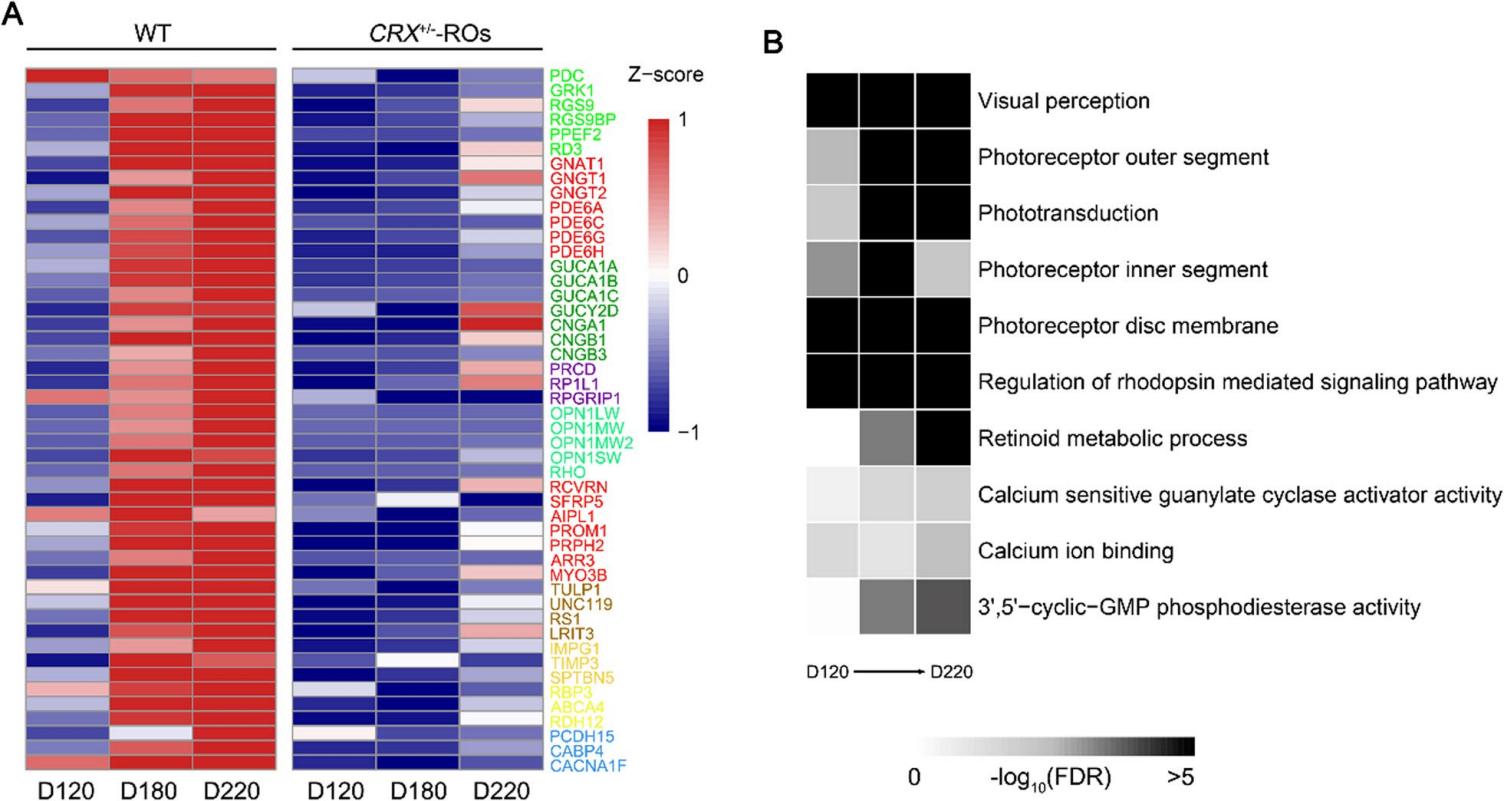
Transcriptomic analysis in wildtype and *CRX*^±^-ROs. **A** Heatmap comparing expression of typical genes across wildtype and *CRX*^±^-ROs at D120, D180 and D220. Values represented the averages of 3 samples in all stages of *CRX*^±^-ROs, and 2 in all stages of Control after quality assessment of RNA sequencing. **B** The Gene Ontology annotation analysis of differentially expressed genes (DEGs) between wildtype and *CRX*^±^-ROs

In order to dissect gene transcriptome changes along with photoreceptor OS/IS absence, we performed RNA-seq analysis of D120, D180 and D220 ROs (Fig. 2). We found that most of the genes downregulated in *CRX*^±^ ROs of these stages were associated with Gene Ontology terms of “visual perception”, “photoreceptor outer segment”, “phototransduction”, “regulation of rhodopsin mediated signaling pathway” and other visual related processes (Fig. 2B). Opsin genes (*OPN1LW, OPN1MW, OPN1MW2, OPN1SW, RHO*) were significantly downregulated in all three differentiation stages, which was consistent with the results of immunostaining. The expression of guanylate cyclase activator genes (*GUCA1A, GUCA1B, GUCA1C*) and phosphodiesterase genes (*PDE6A, PDE6C, PDE6G, PDE6H*) in phototransduction, cell–cell adhesion and skeleton-related genes (*RS1, IMPG1, TIMP3, SPTBN5*), retinoic acid metabolism and calcium-dependent biological processes related genes (*RBP3, ABCA4, RDH12, CABP4*), and other phototransduction regulator genes (*GRK1, PPEF2*) was also decreased at D180 and D220 organoids. These downregulated genes involve in photoreceptor maturation and functional performing, partially overlapping with altered genes in *Crx* mutant mice (13,25).

On the other hand, a small set of downregulated genes at D180 were subsequently upregulated to some extent at D220. These included the genes for catalyzing the synthesis of cyclic GMP in the photoreceptors *(GUCY2D)*, for synaptic transmission between cones and bipolar cells (*LRIT3*), and for calcium sensor to light in photoreceptor (*RCVRN*). Interestingly, the photoreceptor transducin (*GNAT1, GNGT1*) and cyclic nucleotide gating ion channel (*CNGA1, CNGB1*), which were presented in rod cell phototransduction, were partially recovered at D220. However, *GNGT2*, *CNGB3*, *ARR3*, and other important genes in cone development did not recover in D220 organoids. The different performances in rod and cone maturation after *CRX* monoallelic deletion indicate that *CRX* runs different rules in two types of cells.

In addition, we found a set of genes related to cell cycle were upregulated in D180 *CRX*^±^-ROs (Additional file 2: Fig. S2). They involve in a series of biological processes accompanying mitosis, such as centromeric sister chromatid cohesion, mitotic nuclear division, spindle organization and cell cycle phase progress. We speculate that certain types of cells in organoids may go through excessive mitosis at this stage.

### More Müller cells were generated at late differentiation of ***CRX***^±^-ROs

**Fig. 3:**
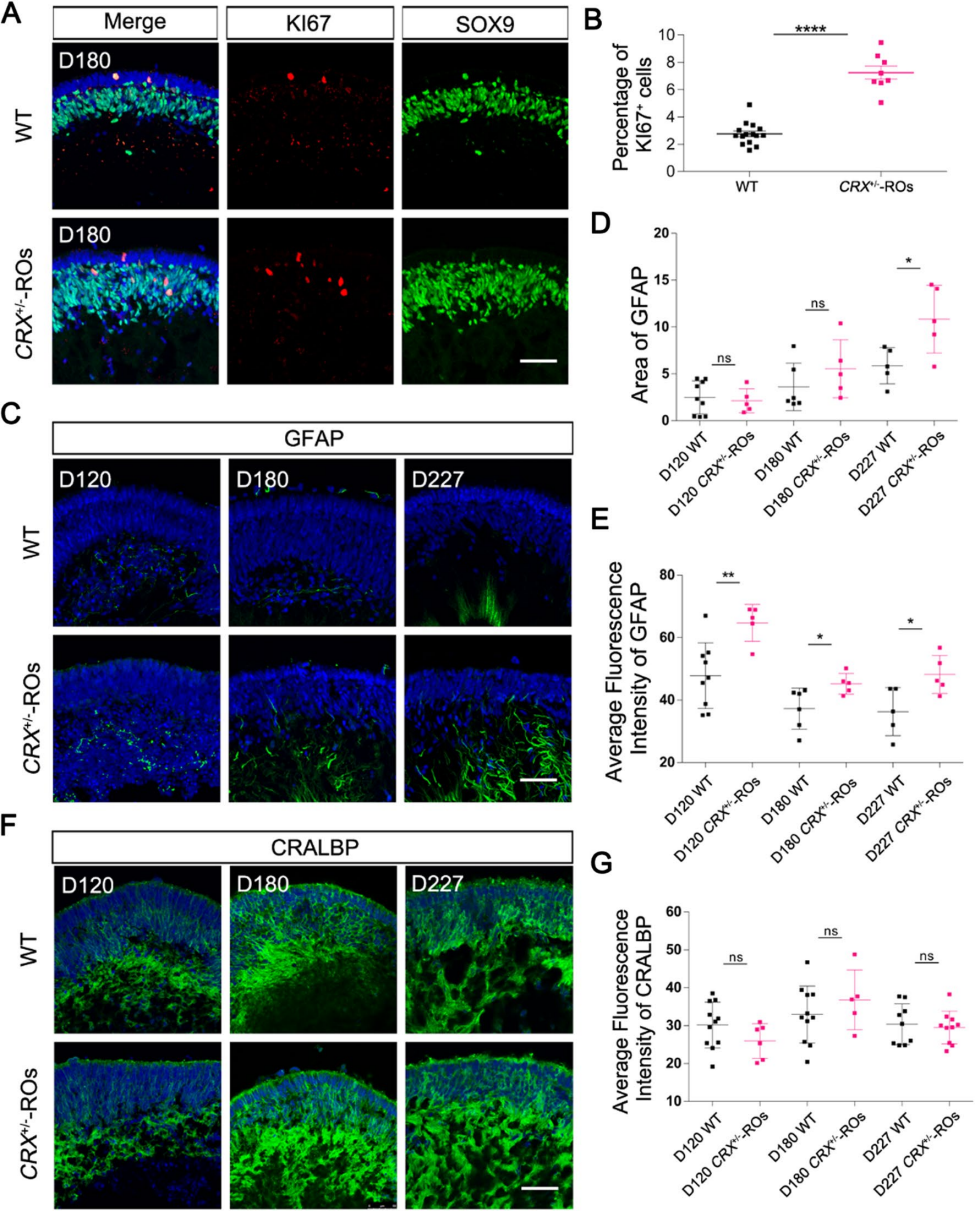
Generation of more Müller cells in *CRX*^±^-ROs at late stage of differentiation. **A** Immunostaining with anti-KI67 and anti-SOX9 in D180 *CRX*^±^-ROs and Control. Scale bar, 50 μm. **B** Quantification of anti-KI67^+^ cells showing significant cell proliferation in D180 *CRX*^±^-ROs. **C** Immunostaining with anti-GFAP in D120, D180 and D227 *CRX*^±^-ROs and Control. Scale bar, 50 μm. **D** Quantification of GFAP^+^ area between *CRX*^±^-ROs and Control. **E** Quantification of average fluorescence intensity of anti-GFAP between *CRX*^±^-ROs and Control. **F** Immunocytochemistry with anti-CRALBP in D120, D180 and D227 *CRX*^±^-ROs and Control. Scale bar, 50 μm. **G** Quantification of average fluorescence intensity of anti-CRALBP between *CRX*^±^-ROs and Control

To examine the proliferative cell types in D180 organoids, we performed immunofluorescence staining of KI67, a pan-proliferation cell marker. KI67^+^ cells existed in both wildtype and *CRX*^±^-ROs, and most of them located in the inner nuclear layer (INL) of organoids (Fig. 3A). All KI67^+^ cells were also SOX9^+^ (a marker of retina progenitors and Müller cells at the stage). The percentage of KI67^+^ cells (7.279 ± 0.4827) in *CRX*^±^-ROs was significantly increased compared with that (2.673 ± 0.2089) of control (Fig. 3B), indicating an extra cell proliferation in late retinal progenitor cells or Müller precursor cells.

For further cell type identification, we examined glial fibrillary acidic protein (GFAP), a marker that is mainly present in the activated Müller end feet and upregulated in retinal injury. Immunofluorescence staining for GFAP showed a punctate or discontinuous fibrillar distribution in D120 organoids, with a marked increase in coarse fibrillar-like structures as differentiation continued, particularly in *CRX*^±^-ROs (Fig. 3C). The GFAP^+^ area increased in D227 *CRX*^±^-ROs (Fig. 3D), and the average fluorescence intensity of GFAP^+^ areas was significantly enhanced in D120, D180 and D227 *CRX*^±^-ROs compared with WT (Fig. 3E), suggesting an increase in activated Müller cells in *CRX*^±^-ROs. However, the cellular retinaldehyde binding protein (CRALBP), a marker widely expressed in the cytoplasm of mature Müller cells, did not enhance their average fluorescence intensity at all timepoints examined (Fig. 3F and G), implying that the number of mature Müller cells was unaffected in *CRX*^±^-ROs.

### Temporary abnormal distribution of CRX^+^ cells during early differentiation of ***CRX***^±^-ROs

**Fig. 4:**
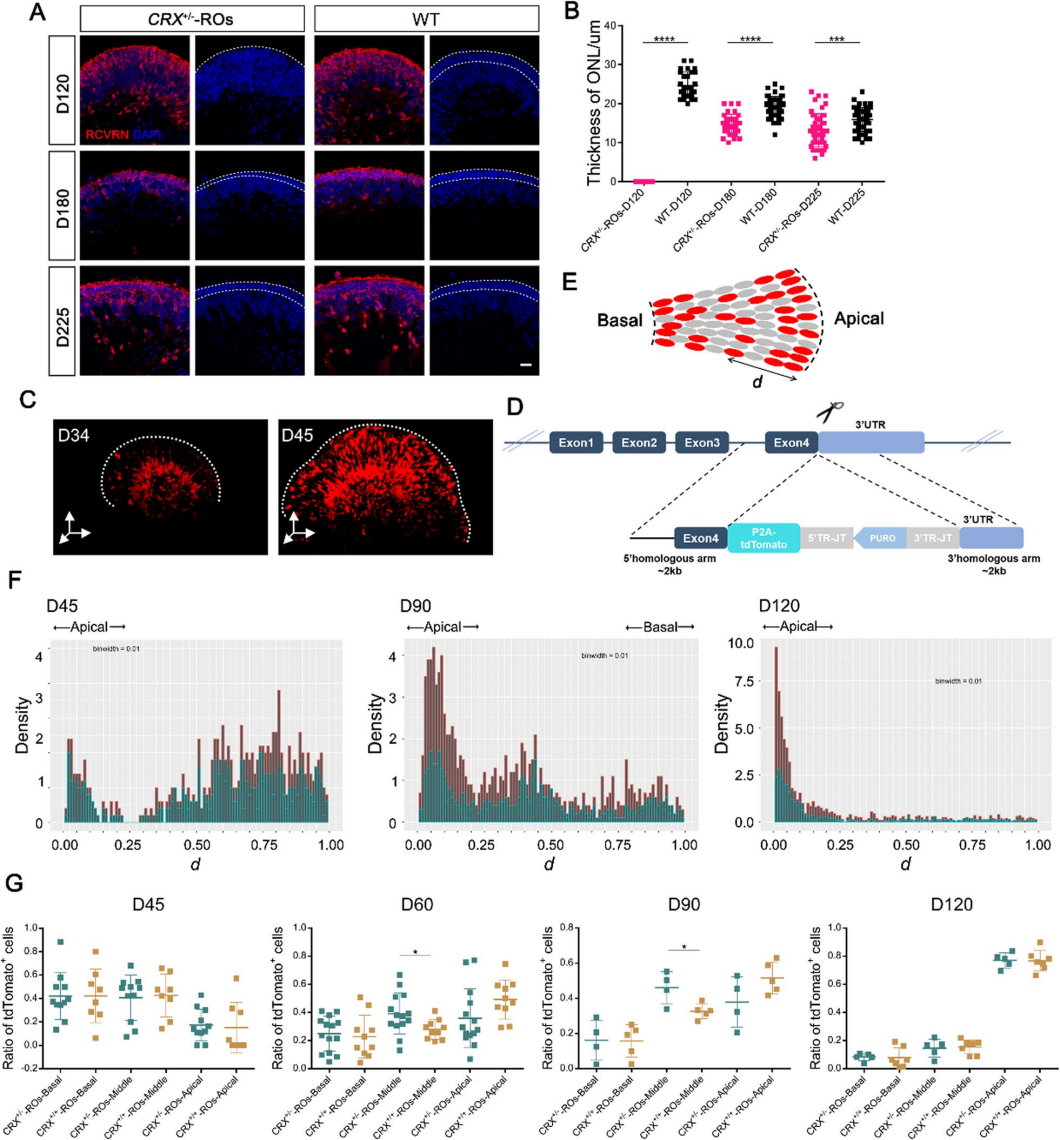
Differential spatial distribution of postmitotic cells between *CRX*^±^-ROs and *CRX*^+*/*+^-ROs. **A** The ONL stratification at the indicated stages in two groups. Immunocytochemistry of Anti-RCVRN and DAPI was used to exhibit ONL formation. Scale bar, 50 μm. **B** Quantification of ONL thickness at the indicated stages between two group. Significance tested with *t*-test: ***, *p* < 0.001; ****, *p* < 0.0001. **C** Two-photon microscopy showing the overview of tdTomato^+^ cell distribution in *CRX*^±^-ROs. **D** Schematic diagram of the construction of *CRX*^+*/*+^ hESCs. **E** Schematic diagram of tdTomato^+^ cell distribution in apical, middle or basal regions.** F** The probability density histogram showed the overall spatial distribution pattern of tdTomato^+^ cells in D45, D90 and D120 *CRX*^±^-ROs and *CRX*^+*/*+^-ROs. Data were displayed by setting the binwidth to 0.01. Green color stands for *CRX*^±^-ROs and brown for *CRX*^+*/*+^-ROs. **G** Quantification of tdTomato^+^ cell percentages at the indicated stages in demarcated regions. Significance tested with *t*-test: *, *p* < 0.05

We have observed a delayed ONL stratification in *CRX*^±^-ROs (Fig. 1C). The ONL stratification is a complex process precisely orchestrated photoreceptor development, migration and synaptic growth, still with many unsolved mysteries [37]. To quantify abnormalities in ONL stratification, we measured ONL thickness in two groups. The results indicated that the ONL in *CRX*^±^-ROs was thinner than that of control at all stages (Fig. 4A and B). The CRX^+^ cells represent the development of postmitotic precursors into photoreceptors, which should eventually translocate into the ONL during development. A careful assessment of the positional information of the CRX^+^ cells is therefore necessary. For this purpose, the degree to which the fluorescent signal (tdTomato) matched the pixels of CRX was firstly checked. We verified the complete colocalization of CRX expression with tdTomato (Additional file 3: Fig. S3A), indicating that the tdTomato reporter could faithfully represent CRX^+^ cells. Two-photon microscopy characterized the distribution of tdTomato^+^ cells in the whole organoids, which showed an initial basal distribution and a gradual broadening to the apical side (Fig. 4C).

As a control, another tdTomato reporter stem cell line was constructed with an alternative knock-in strategy that keeps biallelic *CRX* expression as well as concurrent tdTomato expression with the P2A sequence as a spacer (Fig. 4D). In the following retinalization, the tdTomato^+^ cells emerged in these *CRX*^+*/*+^ organoids (*CRX*^+*/*+^-ROs) around 28 days and gradually increased with differentiation continues, same as that of *CRX*^±^-ROs (Additional file 4: Fig. S4A). We analyzed the number of tdTomato^+^ cells in two groups. Flow cytometric analysis showed considerable consistency in D45, D60 and D90 organoids with positive cell rates of (5.17, 13.8, 32.5) and (5.63, 12.6, 31.8) in *CRX*^±^-ROs and *CRX*^+*/*+^-ROs, respectively (Additional file 4: Fig. S4B and C). Colocalization analysis exhibited a well follow of CRX with tdTomato in *CRX*^+*/*+^-ROs (Additional file 3: Fig. S3B). The expression of *PAX6, RAX, VSX2, SOX2, OTX2* at D45 exhibited no difference in wildtype H9*-*ROs and *CRX*^+*/*+^-ROs (Additional file 5: Fig. S5A). The ONL appeared as expected in *CRX*^+*/*+^-ROs with unaffected brush structure (Additional file 5: Fig. S5B). The results indicated that the tdTomato knock-in cell line does not affect its differentiation pattern to retinal organoids.

To quantify spatial distribution of CRX^+^ cell in organoids, we calculated the relative distance between fluorescent cells and apical of organoids in *CRX*^±^-ROs and *CRX*^+*/*+^-ROs (Fig. 4E). Probabilistic distribution analysis was performed to depict spatial distribution of tdTomato^+^ cells in both groups according to the distance. The probability density histogram showed that most of cells located at basal side in D45 organoids with including small number of cells adjacent to the apical in both groups (Fig. 4F). All tdTomato^+^ cells translocated at apical side in D120 organoids, while D90 organoids showed a transitional state with apical, basal and middle distribution in both groups. Changes in the distribution pattern of tdTomato^+^ cells display the location requirements of photoreceptor development during organoid differentiation.

According to the probability distribution, both groups exhibited fairly definite boundary of apical and basal. We demarcated the cells in the interval for statistics (Apical, 0–0.25; Basal, 0.75–1; Middle, 0.25–0.75). The D60 and D90 organoids showed that there is lower ratio of tdTomato^+^ cells at the apical and a higher ratio at the middle in *CRX*^±^-ROs compared with *CRX*^+*/*+^-ROs (Fig. 4G), suggesting that the CRX^+^ cells encountered a certain degree of translocation obstacles during early differentiation (i.e., before D90). The ratios were restored to the same level in D120 organoids. Cells distribution correction, but not the ONL formation as expected, suggests that the temporary disruption of the postmitotic cell position is attribute to a delay in ONL stratification.

### Live image recording of postmitotic cell translocation in ***CRX***^±^-ROs and ***CRX***^+***/***+^-ROs

Live-cell imaging provides us a new dimension beyond omics to trace the processes of organ development and disease occurrence. In order to investigate the translocation obstacles of CRX^+^ cells, we anchored D60 organoids to perform live-cell imaging. In order to minimize photobleaching and phototoxicity, a dose of laser as small as possible (0.2% of total power) was irradiated in *CRX*^+*/*+^-ROs and *CRX*^±^-ROs. During 28 h of continuous imaging, most of the tdTomato^+^ cells changed their positions in organoids (Additional file 6: Video S1; Additional file 7: Video S2). Cells translocated with elongated morphologies in a pause-motion alternate manner, which may be hindered by the narrow intercellular spaces.

**Fig. 5:**
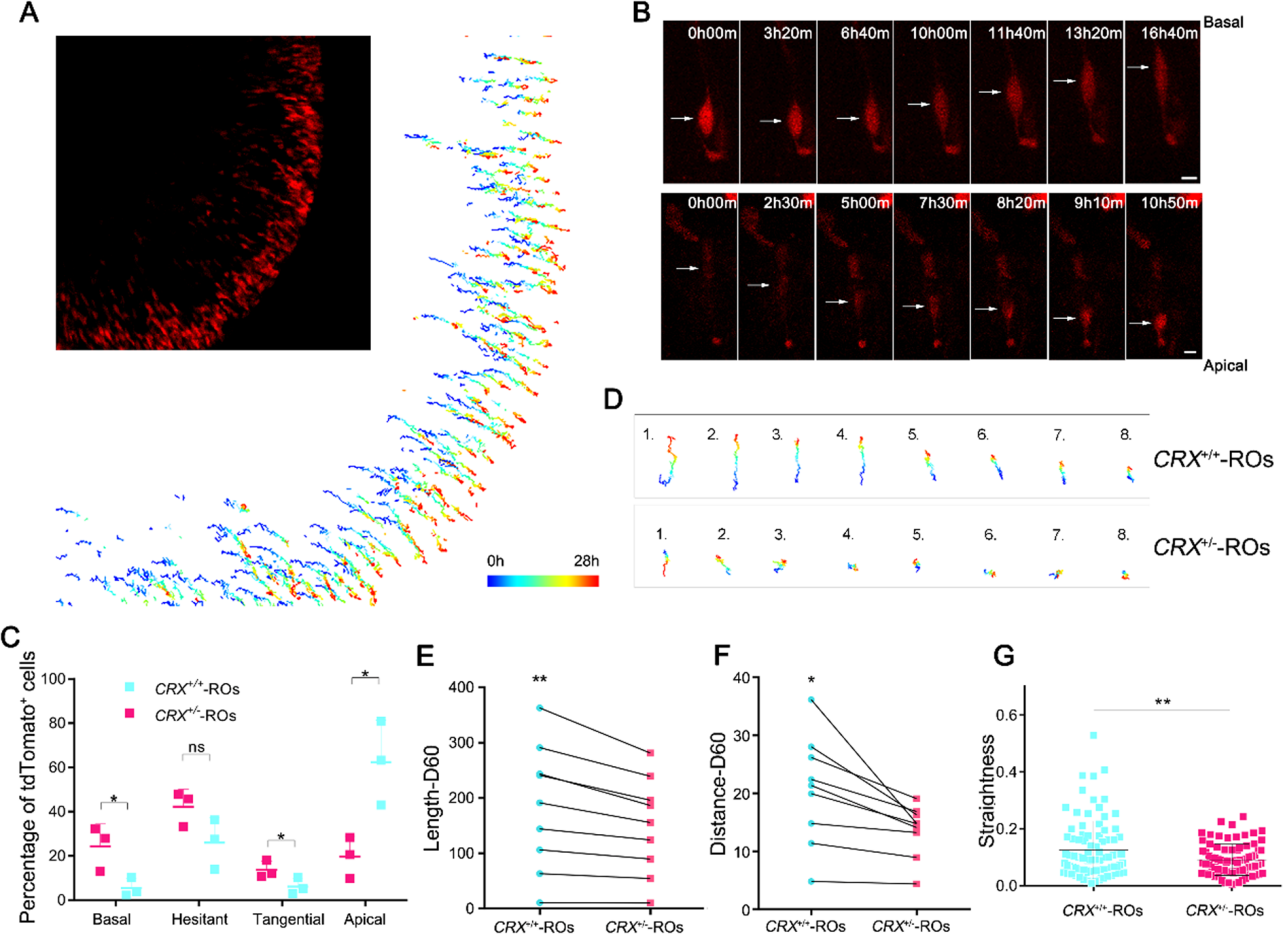
Live-cell imaging of D60 *CRX*^±^-ROs and *CRX*^+*/*+^-ROs. **A** Overview of cell trajectories in the imaging area of *CRX*^+*/*+^-ROs. The color represents cell displacement over time with the live-cell imaging. **B** Representative images of basal- and apical-translocating cells. Scale bar, 5 μm. **C** Quantification of basal-, apical-, tangential-orientated and hesitant cells in *CRX*^±^-ROs and *CRX*^+*/*+^-ROs. Significance tested with *t*-test: *, *p* < 0.05; ns, not significant. **D** Representative cell trajectories in *CRX*^±^-ROs and *CRX*^+*/*+^-ROs.** E** Groups of paths were summed according to the total length of movement.** F** Groups of paths were summed according to distance between start and end positions. Significance tested with paired *t*-test: *, *p* < 0.05; **, *p* < 0.01. **G** Quantification of straightness between two groups. Significance tested with *t*-test: **, *p* < 0.01

To quantify the translocation, we depicted cell trajectories within the same sample thickness after image acquisition (Fig. 5A). We found that the direction of cell translocation is not uniform (Fig. 5B). In addition to some cells that did not move effectively, the other cells either translocated along apical-basal axis or perpendicular to the axis. In *CRX*^+*/*+^-ROs, about 60% of the cells translocated toward the apical side, while this proportion was only about 20% in *CRX*^±^-ROs (Fig. 5C). On the contrary, cells toward basal side and tangential direction were significantly increased in *CRX*^±^-ROs. The number of cells with invalid paths did not show a significant difference between two groups. Selected typical cell trajectories showed different translocation characteristics between two groups (Fig. 5D).

To compare the translocation parameters of tdTomato^+^ cells, we grouped all cells into 9 sets according to time-length of each cell appeared in images for further differential analysis. Distance is used to represent the effect translocation between the start and end positions, while Length is used to sum all paths traveled by a cell (Fig. 5E and F). Both Distance and Length in the *CRX*^±^-ROs were shorter than those in *CRX*^+*/*+^-ROs, showing a relatively inefficient translocation pattern. We further analyzed the straightness of cell paths, which exhibiting significantly lower in *CRX*^±^-ROs than in *CRX*^+*/*+^-ROs. To sum up, CRX^+^ cells showed an unclear direction and relatively inefficient translocation due to *CRX* monoallelic transcription.

### Overtension of actomyosin network restricted postmitotic translocation in ***CRX***^±^-ROs

**Fig. 6:**
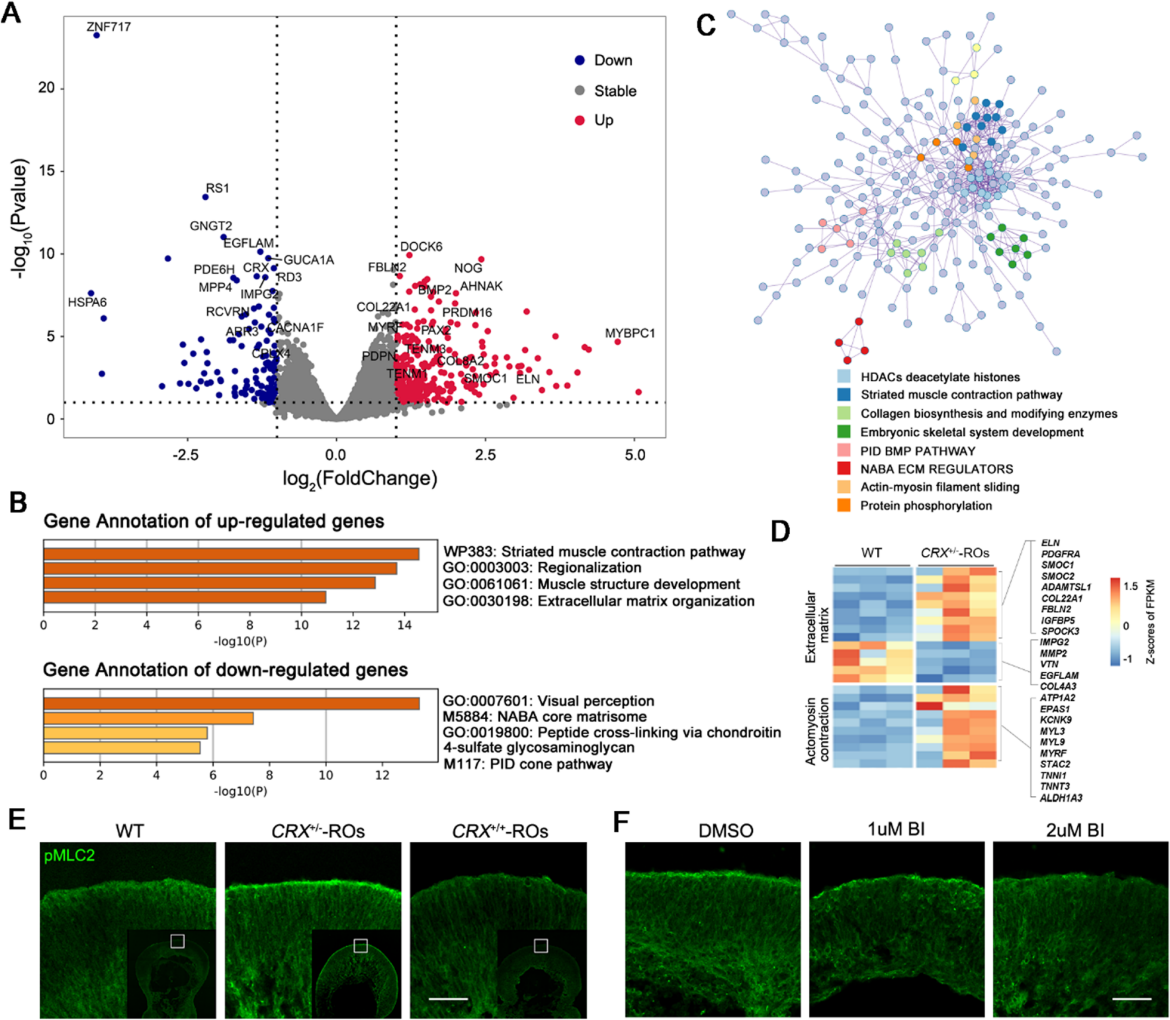
Overtension of actomyosin network in D60 *CRX*^±^*-*ROs and candidate regulators. **A** Differentially expressed genes (DEGs) between the *CRX*^±^-ROs and the wildtype group. The red plots represent the upregulated genes and the blue plots represent the downregulated genes. **B** Gene enrichment annotation analysis of DEGs and the Top 4 terms in up- and downregulation, respectively. **C** Protein–protein interaction analysis by DEGs and the Top 8 clusters. **D** Heatmaps depict Z-scores of FPKM of genes related to actomyosin contraction and extracellular matrix. **E** Immunostaining of pMLC2 in *CRX*^+*/*+^-, *CRX*^±^-ROs and wildtype groups. Scale bar, 50 μm. **F** Immunostaining of pMLC2 in *CRX*^±^-ROs with blebbistatin. BI: blebbistatin. Scale bar, 50 μm

To further investigate changes in underlying molecules and pathways to restrict translocation of CRX^+^ cells, we performed RNA-seq analysis of D60 organoids in *CRX*^±^-ROs and wildtype group. When setting significance threshold corresponding to an adjusted *p* value < 0.05, we obtained 118 upregulated and 58 downregulated differentially expressed genes (DEGs) in *CRX*^±^-ROs. Among which, we focused on genes with larger foldchanges, most significant differences, with higher expression levels (Fig. 6A). Genes of extracellular matrix (*ELN, COL8A2, COL22A1, TENM3 and FBLN2*), signal molecules and transcriptional regulators related with retina development (*NOG, BMP2, PRDM16, PAX2, BMPR1B and SMOC1*), cell adhesion and cytoskeleton (*AHNAK, MYBPC1, PDPN, DOCK6 and TENM1*) were significantly upregulated. In contrast, genes related to visual development (*RCVRN, RD3, RS1, PDE6H, ARR3, GUCA1A, IMPG2, GNGT2 and MPP4*), multifunction cellular process (*ZNF717, HSPA6*) and synaptic development and transmission (*EGFLAM, CPLX4*) were significantly downregulated.

To evaluate biological processes more systematically, we performed enrichment analyses on DEGs. For this purpose, we set significance threshold corresponding to a *p* value < 0.1, resulting in an enlarged set of DEGs with 316 upregulated and 134 downregulated. The top 4 upregulated genesets were associated with “striated muscle contraction pathway”, “regionalization”, “muscle structure development”, and “extracellular matrix organization”. The top 4 downregulated genesets were involved with “visual perception”, “NABA core matrisome”, “peptide cross-linking via chondroitin 4-sulfate glycosaminoglycan”, and “PID cone pathway” (Fig. 6B). Meanwhile, the simulated molecular complexes in protein–protein interaction analysis showed that there were 4 clusters contributing to muscle contraction pathway and extracellular matrix regulation in the top 8 dense regions (Fig. 6C). Previous studies have shown that actomyosin contractility and extracellular matrix organization affect multiple dynamics, such as migration, cell shape, cell division and tissue morphogenesis. Therefore, it is not surprising that actomyosin contraction and extracellular matrix-related genesets were present in downstream of *CRX* (Fig. 6D), suggesting that they became potential regulators involved in the abnormal translocation of photoreceptor precursors in *CRX*^+*/*+^-ROs. The data probably indicated a differentially activated actomyosin network between two groups.

Actin interacts with molecular motor myosin and crosslinkers to generate contractility. This activated process is energized by ATPase hydrolysis and characterized by myosin phosphorylation. To verify whether there was an overactivated actomyosin network, we examined the levels of phosphorylated myosin light chain 2 (pMLC2), a local indicator of actomyosin activation, in wildtype*, CRX*^±^*-* and *CRX*^+*/*+^-ROs (Fig. 6E). The *CRX*^±^-ROs had a significantly stronger accumulation of pMLC2 especially near the spherical surface than the other two groups.

Blebbistatin, a selective small molecule inhibitor of motor myosin, can slow down phosphate release in ATPase catalysis. We found that blebbistatin attenuated the accumulation of pMLC2 (low concentration, ≤ 2 μM) on the spherical surface of *CRX*^±^-ROs in a dose-dependent manner (Fig. 6F), confirming the presence of an overactivated actomyosin network in *CRX*^±^-ROs. The stronger contractility suggested that the overtension network in *CRX*^±^-ROs impeded the translocation of photoreceptor precursor cells.

## Discussion

Elucidation of pathogenic mechanism of gene mutations helps to develop therapeutic strategies. In this study, we established a monoallelic deletion of *CRX* in hESCs and demonstrated that *CRX* haploinsufficiency caused severe photoreceptor maturation impairment using retinal organoid differentiation. We further demonstrated that the overtension of actomyosin network impeded efficient translocation of photoreceptor precursor cells in early retinal differentiation and delayed the ONL stratification. These results provided a new mechanism of retinal degeneration caused by *CRX* haploinsufficiency.

A mild phenotype appeared in *Crx*^±^ heterozygous knockout mice with slightly shorter OSs at P14 and a gradual recovery of ERG amplitude with retinal development. While homologous *Crx*^*−/−*^ resulted in a reduced ONL thickness, loss of the OSs, and complete disappearance of ERG response in mouse retina [38]. In human RO differentiation, we demonstrated that *CRX* monoallelic transcription resulted in thinner ONL thickness, extensive absence of OSs, and downregulation of genes for OS formation, phototransduction and photoreceptor cell development, which were more similar to phenotypes in *Crx*^*−/−*^ mice. Such phenotypic differences between human and mouse possibly reflect the heterogenicity of gene regulatory networks across species. On the other hand, clinically heterozygous mutations with complete or nearly complete abolish of *CRX* monoallelic function caused mild or unaffected visual acuity in addition to severe phenotypes [21, 22, 24]. Therefore, there are other transforming factors involved in the heterogenicity of *CRX* mutations which could explain disease occurrence. Even though, photoreceptor immaturity due to *CRX* haploinsufficiency should be considered as a nonnegligible pathogenic factor for patients with severe visual impairment, and gene augmentation rescue may be an effective treatment strategy.

We also observed an increase in Müller cells by enhanced proliferation of late retinal progenitor cells and/or Müller precursor cells in the late stage of *CRX*^±^-ROs differentiation. Normal physiological function of Müller cells is essential for maintaining retinal integrity and function. Its proliferation is usually accompanied by upregulation of glial fibrillary acidic protein (GFAP) and vimentin, which is also associated with retinal damage and gliofibrosis. In recent years, Müller cells have been regarded as a renewable resource for mammalian retinal repair [39, 40]. Müller cells can protect photoreceptor cells through releasing neurotrophic factors and antioxidants to surrounding microenvironment when a programmed cell death occurring [39, 41]. However, when Müller glial cells were stimulated to proliferate to a large amount, they usually committed to epithelium-to-mesenchymal transition and gliofibrosis and filled the space left by the dead photoreceptor cells, which eventually led to substantial inhibition of photoreceptor cell regeneration [41]. In this study, upregulation of Müller cells is observed at the cost of reduction in photoreceptor cells during *CRX*^±^-ROs development; however, the effect of this proliferation needs to be further evaluated.

Cells coordinate their own shape through the formation of an actomyosin contractile network that is composed of actin filaments and myosin motors, thereby participating in important biological processes including cell division and cell migration [42]. This network is tightly regulated in space and time, and its abnormal activation could lead to metastasis of tumor cells and blockage of neuronal extension [43–46]. Elevation of pMLC is the hallmark event of network activation. At early stage of self-organized retinal organoids derived from stem cells, actomyosin activity provides differential mechanical rigidity between RPE cells and neural retina, ensuring the generation of optic cups similar to embryonic development [47–50]. As differentiation continues, neural progenitor cells exit their cell cycles to complete fate specialization; typical stratified neural structures are formed to build neural connections. It is still unknown whether abnormal actomyosin activity affects neural differentiation during this process.

The lack of literature description in previous studies may be due to its speculative low network intensity. In reality, the higher intensity of actomyosin network provides stiffer environment that affects the division and differentiation of neural progenitor cells [51]. Therefore, we reason that normal intensity of actomyosin network may be necessary to neural retina development. In this study, *CRX* monoallelic transcription created a relatively more active actomyosin in the entire organoid, ultimately blocking effective translocation of postmitotic photoreceptor cells and delaying outer retina nuclear layer formation. Actomyosin may not directly involve in postmitotic photoreceptor cell translocation, which has been proved to be intrinsically driven by nuclear migration [52, 53], but dynamically influences the efficiency of cell translocation through the construction of the external environment.

The location of neural cell production during retinal development is often not the same as the location where they function. They complete migration, layering, and establish precise neural connections to receive, process and transmit light signals [54–56]. Prompt and accurate cellular localization is essential to ensure establishment of correct neural connections to exert visual function [54, 57, 58]. In D120 *CRX*^±^-ROs, the ONL stratification still did not appear as expected, but required a longer time to form, confirming the stringent prerequisite conditions for retinal stratification. Imperfect temporal and spatial matching of cell status may affect other cells in retina and thus, caused a cascade amplification to complicate the result of photoreceptor defects. In future, timely drug intervention for cell translocation plus gene augmentation rescue may be a better treatment strategy for *CRX* mutation diseases.

In this study, we simulated retinal development with a monoallelic knockout of *CRX* in retinal organoid differentiation, which gave us the opportunity to study the formation of the human retina in a unique way. However, the importance of *CRX* is not limited to the photoreceptor development and maturation. Unfortunately, the lack of vascular system in our retinal organoids to transport nutrients inside will eventually trigger inadequate internal metabolism and apoptosis in the long-term culture. In the future, a more refined organoid culture system would help to uncover the long-term effects of monoallelic knockout of *CRX*.

## Conclusion

We differentiated hESCs into retinal organoids to sequentially uncover the phenotypes of *CRX* monoallelic transcription at different stages of development. We confirmed that *CRX* haploinsufficiency caused defective precursor translocation in early retinal development, and that overtension of actomyosin network participated in this process. We provided evidence that haploinsufficiency constituted a dominant pathogenic basis for *CRX* hereditary diseases in patients. This study suggests that the combination of actomyosin tension-modulating drugs with *CRX* augmentation may provide a more precise and effective treatment for *CRX*-associated retinopathies in human.

### Supplementary Information

**Additional file 1**: **Figure S1**. Characterization of CRX+/--ROs. A Representative bright field images of CRX+/--ROs and wildtype ROs at D36, D60 and D90. Scale bar, 400 μm. B Immunostaining of VSX2, OTX2, PAX6, RAX and SOX2 showed no difference between D45 CRX+/--ROs and wildtype ROs. Scale bar, 50 μm.

**Additional file 2**: **Figure S2**. Heatmaps at A D120 and B D180 to show changes of cell cycle related genes in wildtype ROs and CRX+/--ROs.

**Additional file 3**: **Figure S3**. The tdTomato signals were colocalized with CRX+ cells in A CRX+/--ROs and B CRX+/+-ROs. Scale bar, 50 μm.

**Additional file 4**: **Figure S4**. Comparison of tdTomato expression in CRX+/--ROs and CRX+/+-ROs. A Overall expression of tdTomato in D45, D60 and D90 organoids. Scale bar, 400 μm. B Representative flow cytometry analysis in D45, D60 and D90 ROs, respectively. Above: CRX+/--ROs. Below: CRX+/+-ROs. (C) Comparison of increasement tendency of tdTomato+ cells by flow cytometry analysis.

**Additional file 5**: **Figure S5**. Characterization of CRX+/+-ROs. A Immunostaining of VSX2, OTX2, PAX6, RAX and SOX2 showed no difference between D45 CRX+/+-ROs and wildtype ROs. B Representative bright field images of CRX+/+-, CRX+/-- and wildtype ROs at D232, D222 and D225, respectively. Scale bar, 400 μm.

**Additional file 6**: V**ideo S1**. Dynamics of photoreceptor precursors in D60 CRX+/--ROs. A resolution of 1024×1024-pixel images was acquired for about 28 hours with thickness of 30μm to make the maximum projection. Most of the tdTomato-positive cells changed their positions during live imaging. Arrows pointed to significant translocation of cells toward the basal side.

**Additional file 7**: **Video S2.** Dynamics of photoreceptor precursors in D60 CRX+/+-ROs. A resolution of 1024×1024-pixel images was acquired for about 28 hours with thickness of 30μm to make the maximum projection. Most of the tdTomato-positive cells changed their positions during live imaging. The majority of tdTomato-positive cells translocated toward the apical side (shown by arrows), while cells toward the basal side were not clearly visible.

## Data Availability

RNA-Seq data have been deposited in the National Genomics Data Center (NGDC; https://ngdc.cncb.ac.cn/), part of the China National Center for Bioinformation Center (accession number HRA005977).

## Abbreviations

ESCs: Embryonic stem cells
CRX: Cone-rod homeobox
ROs: Retinal organoids
ONL: Outer nuclear layer
INL: Inner nuclear layer
OS: Photoreceptor outer segments
IS: Photoreceptor inner segments
