# Supplementary figures and images for: CRX haploinsufficiency compromises photoreceptor precursor translocation and differentiation in human retinal organoids
Source: Stem Cell Res Ther. 2023 Dec 5;14:346. doi: 10.1186/s13287-023-03590-3 (PMC10696917; doi:10.1186/s13287-023-03590-3)

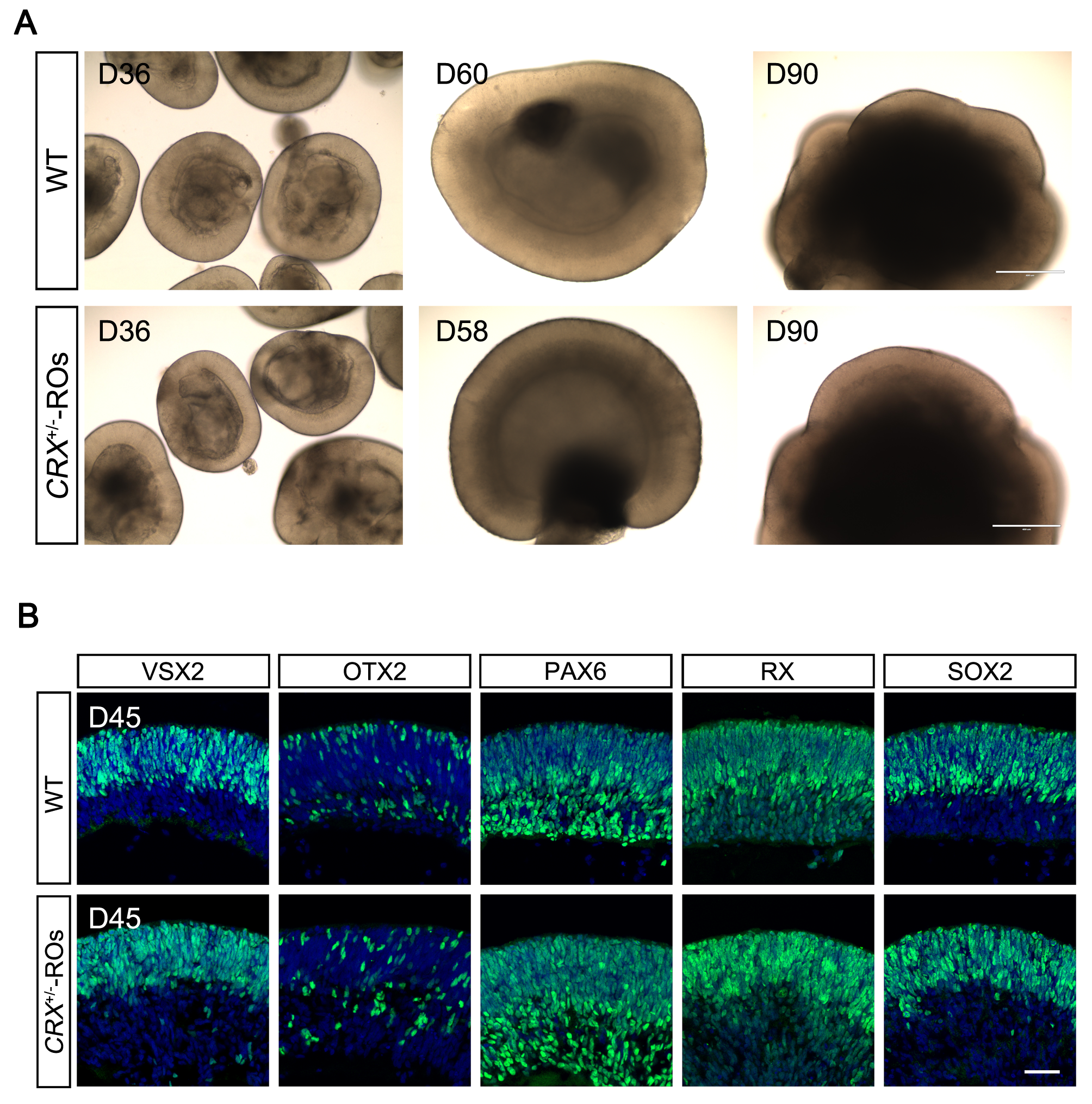

Supplement: Supplementary file 1 — Additional file 1: Figure S1. Characterization of CRX+/--ROs. A Representative bright field images of CRX+/--ROs and wildtype ROs at D36, D60 and D90. Scale bar, 400 μm. B Immunostaining of VSX2, OTX2, PAX6, RAX and SOX2 showed no difference between D45 CRX+/--ROs and wildtype ROs. Scale bar, 50 μm. [file 13287_2023_3590_MOESM1_ESM.tif]

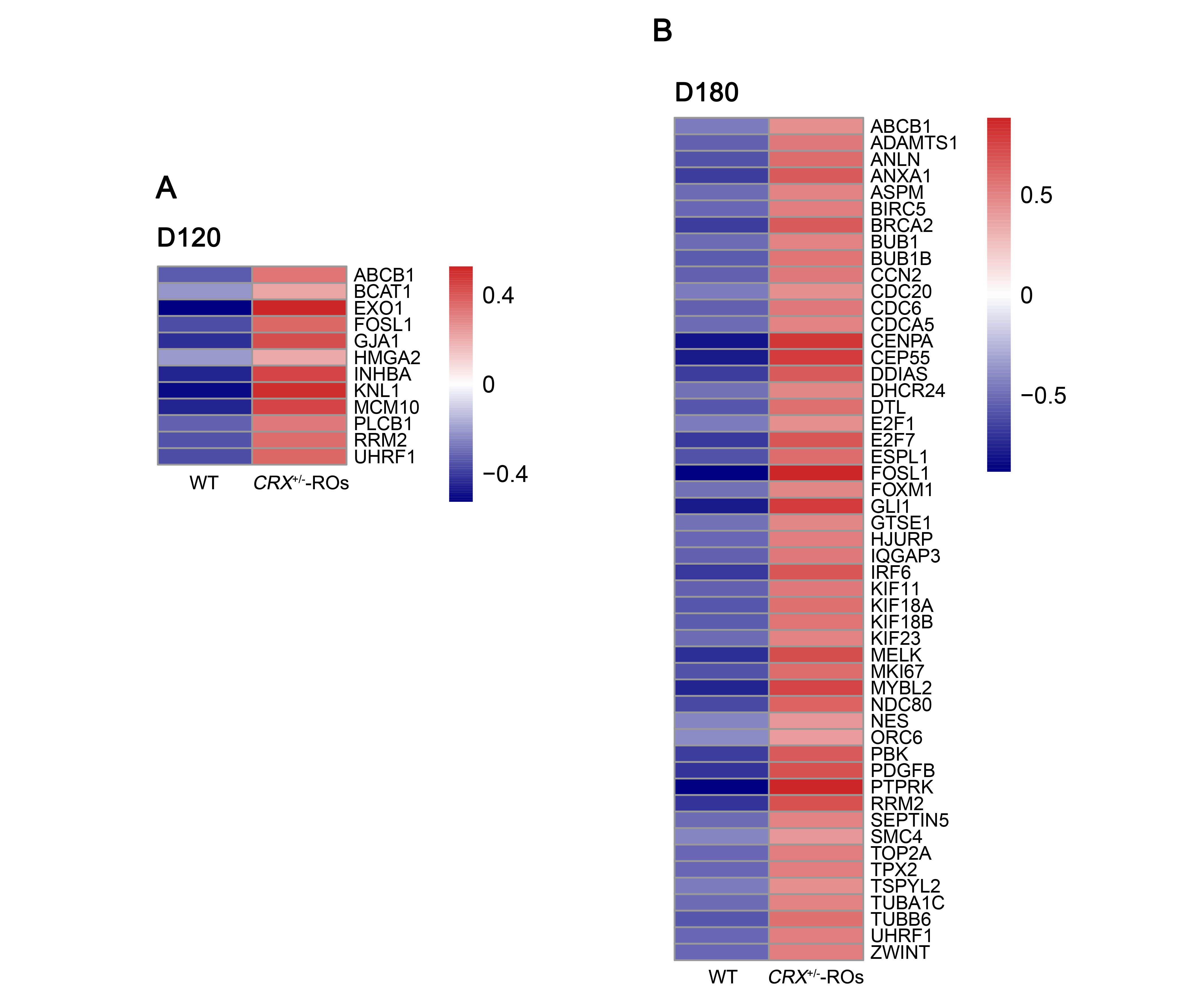

Supplement: Supplementary file 2 — Additional file 2: Figure S2. Heatmaps at A D120 and B D180 to show changes of cell cycle related genes in wildtype ROs and CRX+/--ROs. [file 13287_2023_3590_MOESM2_ESM.tif]

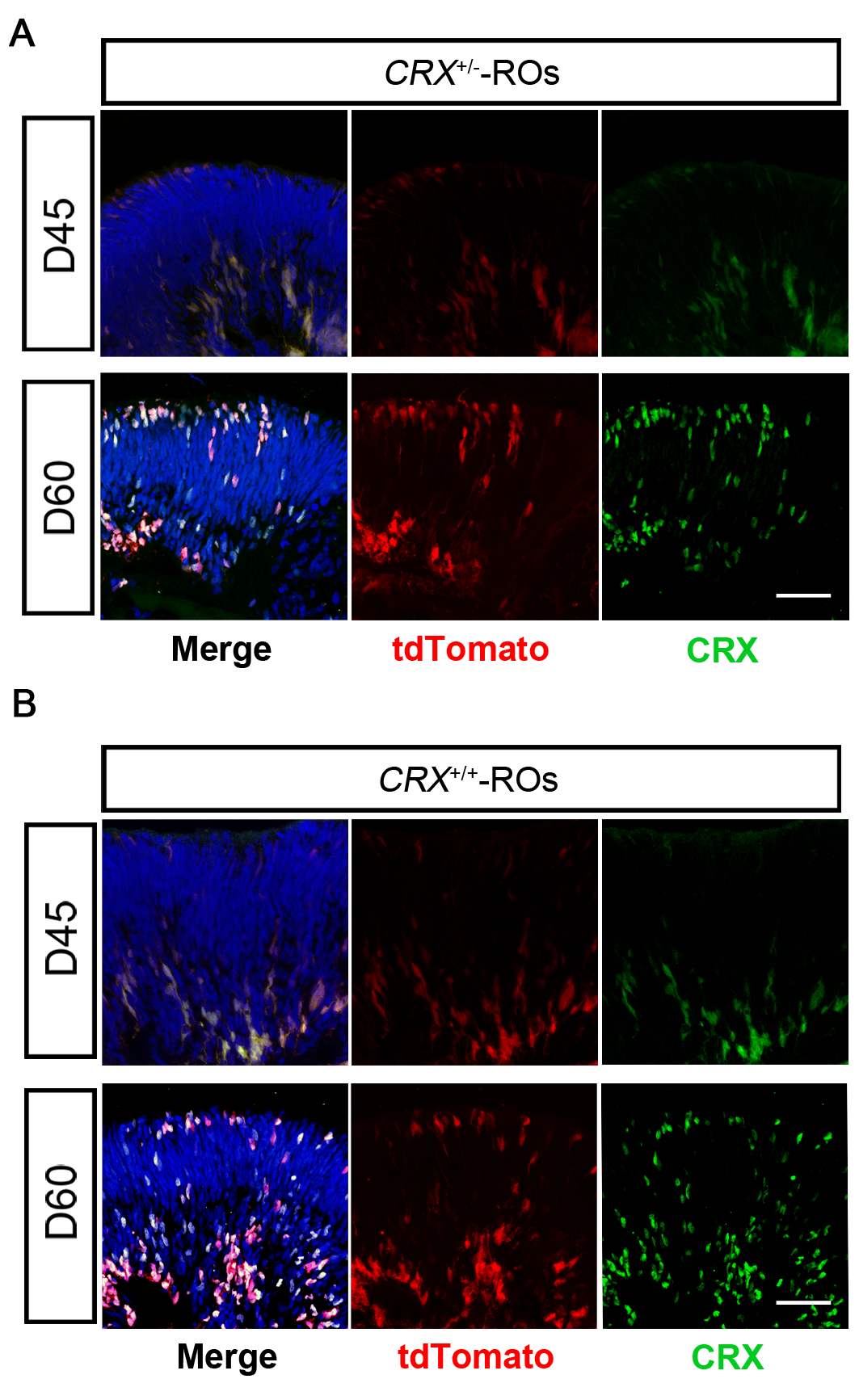

Supplement: Supplementary file 3 — Additional file 3: Figure S3. The tdTomato signals were colocalized with CRX+ cells in A CRX+/--ROs and B CRX+/+-ROs. Scale bar, 50 μm. [file 13287_2023_3590_MOESM3_ESM.tif]

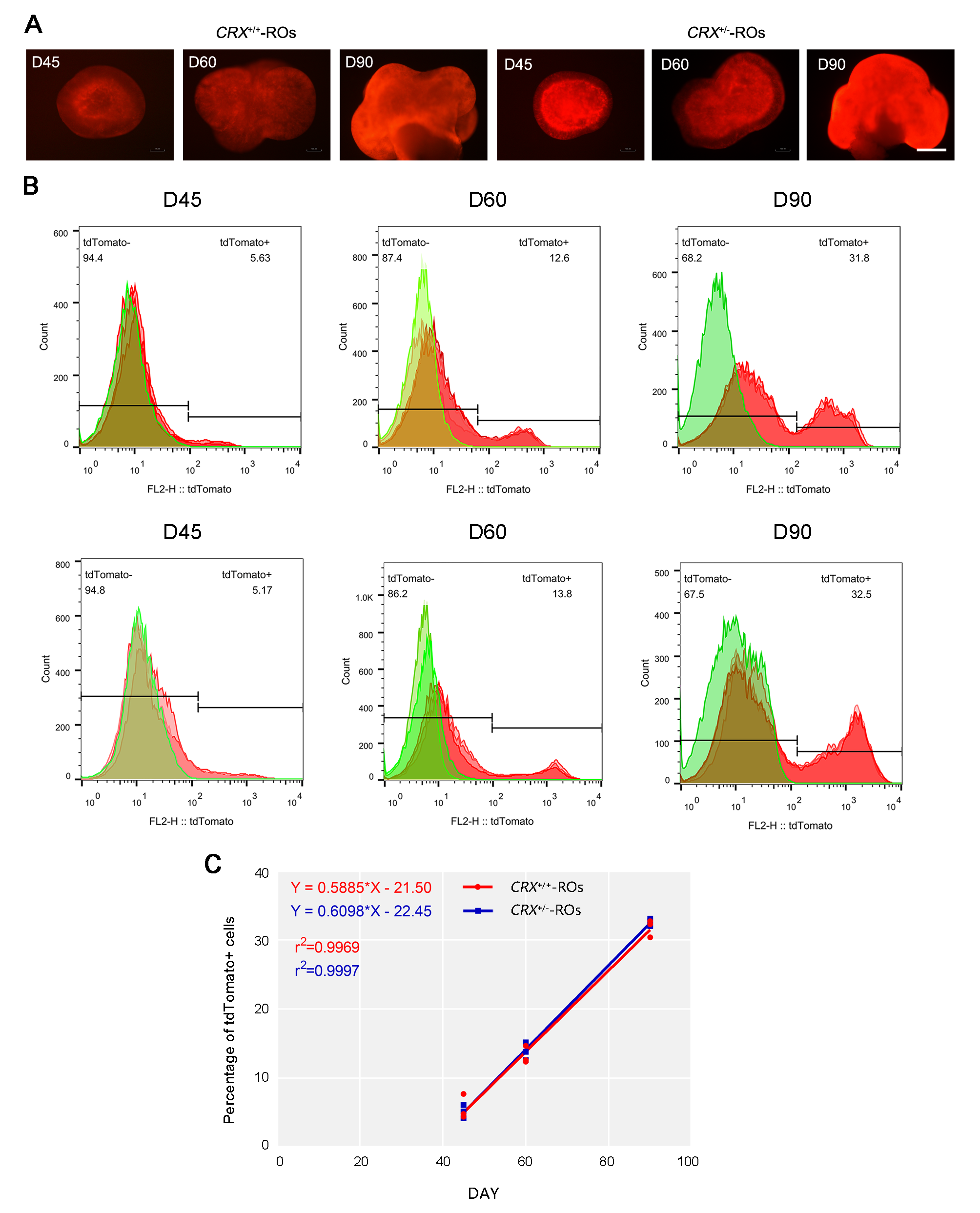

Supplement: Supplementary file 4 — Additional file 4: Figure S4. Comparison of tdTomato expression in CRX+/--ROs and CRX+/+-ROs. A Overall expression of tdTomato in D45, D60 and D90 organoids. Scale bar, 400 μm. B Representative flow cytometry analysis in D45, D60 and D90 ROs, respectively. Above: CRX+/--ROs. Below: CRX+/+-ROs. (C) Comparison of increasement tendency of tdTomato+ cells by flow cytometry analysis. [file 13287_2023_3590_MOESM4_ESM.tif]

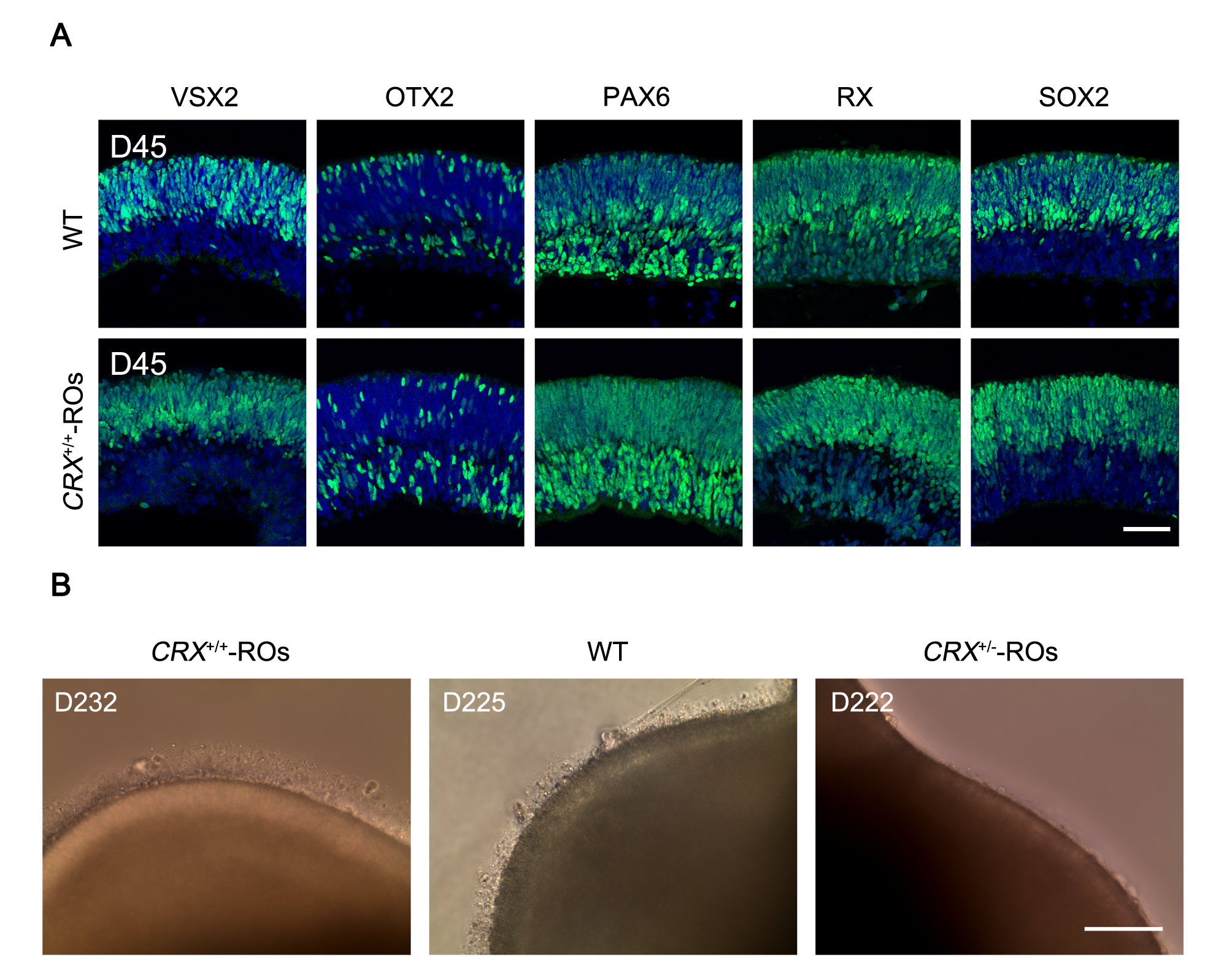

Supplement: Supplementary file 5 — Additional file 5: Figure S5. Characterization of CRX+/+-ROs. A Immunostaining of VSX2, OTX2, PAX6, RAX and SOX2 showed no difference between D45 CRX+/+-ROs and wildtype ROs. B Representative bright field images of CRX+/+-, CRX+/-- and wildtype ROs at D232, D222 and D225, respectively. Scale bar, 400 μm. [file 13287_2023_3590_MOESM5_ESM.tif]
